# Residual Learning and Multi-Path Feature Fusion-Based Channel Estimation for Millimeter-Wave Massive MIMO System

**DOI:** 10.3390/e24020292

**Published:** 2022-02-18

**Authors:** Xuhui Zheng, Ziyan Liu, Jing Liang, Yingyu Wu, Yunlei Chen, Qian Zhang

**Affiliations:** 1 College of Big Data and Information Engineering, Guizhou University, Guiyang 550025, China; zxh1121467573@163.com (X.Z.); ljsrm77715@163.com (J.L.); 18892334373@163.com (Y.W.); 18892332381@163.com (Y.C.); zhangq5065@163.com (Q.Z.); 2 Institute of Computing Technology, Chinese Academy of Sciences, Beijing 100190, China

**Keywords:** millimeter-wave massive MIMO, channel estimation, image super-resolution, residual learning, multi-path feature fusion, dense connection

## Abstract

Channel estimation is a challenging task in a millimeter-wave (mm Wave) massive multiple-input multiple-output (MIMO) system. The existing deep learning scheme, which learns the mapping from the input to the target channel, has great difficulty in estimating the exact channel state information (CSI). In this paper, we consider the quantized received measurements as a low-resolution image, and we adopt the deep learning-based image super-resolution technique to reconstruct the mm Wave channel. Specifically, we exploit a state-of-the-art channel estimation framework based on residual learning and multi-path feature fusion (RL-MFF-Net). Firstly, residual learning makes the channel estimator focus on learning high-frequency residual information between the quantized received measurements and the mm Wave channel, while abundant low-frequency information is bypassed through skip connections. Moreover, to address the estimator’s gradient dispersion problem, a dense connection is added to the residual blocks to ensure the maximum information flow between the layers. Furthermore, the underlying mm Wave channel local features extracted from different residual blocks are preserved by multi-path feature fusion. The simulation results demonstrate that the proposed scheme outperforms traditional methods as well as existing deep learning methods, especially in the low signal-to-noise-ration (SNR) region.

## 1. Introduction

The millimeter Wave (mm Wave) has become one of the research hotspots for its rich bandwidth resources and anti-interference ability in future mobile communication systems [1]. Aiming at solving the problem of path loss in mm Wave, the combination of massive MIMO and mm Wave is used to eliminate the loss by using the high beam fugacity gain provided by large antenna arrays [2]. However, it is difficult to obtain accurate channel state information (CSI) especially in the low SNR region because there is a lot of fading in mm Wave massive MIMO communication systems. In this paper, we focus on the channel estimation approaches for a mm Wave massive MIMO system.

The traditional channel estimation methods mainly include least squares (LS), minimum mean square error (MMSE) [3] and compressed sensing-based algorithms [4,5,6]. However, the assumption that pilot length is larger than the antennas at the BS in the mm Wave massive MIMO system makes channel estimation computationally complicated and creates a huge pilot overhead. In recent years, deep learning (DL) has attracted the attention of researchers in wireless communication fields and has been successfully applied to key physical layer techniques such as modulation pattern recognition [7,8,9,10], blind channel equalization [11], channel decoding [12,13] and channel estimation [14,15,16,17,18,19,20,21,22,23,24,25]. The authors of [14] use powerful deep learning to address the orthogonal frequency division multiplexing (OFDM) system in an End-to-End manner for combating nonlinear distortion and interference. From the simulation results, the DL-based method solves the channel distortion and detects the transmitted symbols with better performance than the LS, but it implicitly estimates the CSI without computing the channel impulse response (CIR). To solve this problem, the work in [15] proposes a deep learning-based image processing technique that considers the time-frequency response of a fast-fading channel as a two-dimensional image and directly estimates the channel matrix by the proposed ChannelNet. The results rival the MMSE but require training multiple networks for different SNR. A deep denoising convolutional neural network (DnCNN) for improving the model’s robustness is proposed in [16], which learns rapidly changing channel characteristics and accurately estimates the channel amplitudes for frequency-selective channel estimation. Motivated by the advantages of residual learning, the studies in [17,18,19,20] introduce a residual learning based estimator, which greatly reduces the implementation complexity. The loss functions for channel estimation are not well designed in a mm Wave massive MIMO system. Therefore, the authors of [21] develop a conditional generative adversarial network (cGAN) to predict more realistic channels by adversarial training. The results show that cGAN is more effective for channel estimation. Inspired by [15], an advanced DL-based super-resolution channel estimation framework EDSR is proposed in [22], and the results in practical 5G simulation environments show that EDSR improves the estimation accuracy and reduces the bit error ratio (BER). Aiming at higher channel estimation accuracy without transmitting longer training sequences, a channel estimation algorithm based on Generative Adversarial Networks (GAN) is proposed in [23], which effectively achieves better estimation performance than that of traditional estimation algorithms. To reduce the pilot overhead in the time-varying cascaded channel estimation over reconfigurable intelligent surface (RIS)-assisted communication, the work in [24] proposes a DL-based channel extrapolation over both antenna and time domains. Specifically, the entire neural network is divided into the recurrent neural network (RNN) and the enhanced feedforward neural network (FNN) to achieve better extrapolation performance. In [25], a two-stage DNN structure with nonlinear modules is proposed to simultaneously generate channel estimation in real-time. The simulation shows that the proposed method is robust to all kinds of nonlinear channel distortion.

With the increasing antennas at the base station (BS) in the mm Wave massive MIMO communication system, severe problems of complex matrix inverse operation and huge pilot overhead are produced. There are some recent works addressing the wave imaging on the machine-learning approach [26,27,28]. Motivated by the methods mentioned above, we regard the quantized received measurements at the BS as a low-resolution image and adopt a state-of-the-art channel estimation framework based on residual learning and multi-path feature fusion to reconstruct the mm Wave channel accurately.

The contributions of this paper are summarized as follows:

- The quantized received measurements and mm Wave channel can be regarded as a low-resolution image and a high-resolution image, respectively. Then, we adopt DL-based image super-resolution techniques to address the non-trivial mapping from quantized received measurements to the mm Wave channel.
- The residual learning is introduced to train only the high-frequency residual part between the quantized received measurements and real mm Wave channel for reducing the training difficulty of the channel estimation model. Furthermore, to prevent the gradient dispersion problem of the estimator due to stacking residual blocks, we conduct a dense connection to ensure maximum information flow between the different layers of the estimator.
- To make full use of the hierarchical features from the quantized received measurements for accurate reconstruction of the mm Wave channel, we perform multi-path local feature fusion and global feature fusion in the estimator.
- We consider the real part and imaginary part as different dimensions of the same image to take advantage of the correlation of the spatial arrangement of the quantized received measurements and the mm Wave channel.

The remainder of this paper is organized as follows. In Section 2, the mm Wave massive MIMO system model is introduced. Section 3 presents the proposed channel estimation scheme based on residual learning and multi-path feature fusion. Correspondingly, the learning strategy for channel estimation and dataset generation is described in Section 4. Section 5 shows the simulation results. Finally, a short conclusion of this paper and future work are summarized in Section 6.

## 2. System Model

**Figure 1:**
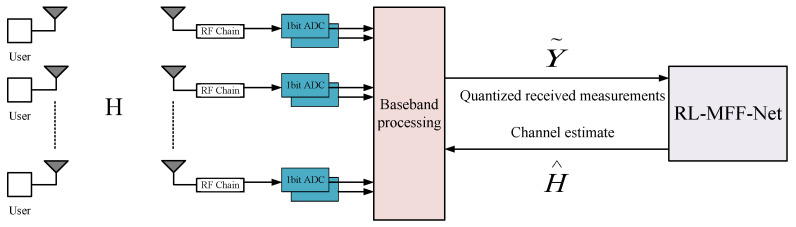
The structure of the system model for channel estimation.

Figure 1 shows a narrowband single-cell mm Wave massive MIMO system with one-bit analog-to-digital converters (ADC), where the BS is equipped with a uniform linear array (ULA) of *M* antennas and $N_{RF}$ RF chains to serve *U* single-antenna users, and U≪M is considered.

The channel between the BS and the *u*-th user can be expressed as

(1) $$h_{u}=\sum_{l=1}^{L}g_{l}^{u}a_{r}\left(\alpha_{l}^{u}\right),$$

where *L* is the number of paths from the users to the BS, $g_{l}^{u}$ and $\alpha_{l}^{u}$ denote the path gain and the angle-of-arrival corresponding to the *l*-th path, respectively. $a_{r}\left(\alpha_{l}^{u}\right)$ refers to the array response of the BS. Therefore, the channel between the BS and the U users is represented as follows

(2) $$H=\left[h_{1},h_{2},\ldots ,h_{u},\ldots ,h_{U}\right].$$

The channel response $H_{v}$ in the angular domain is obtained by performing a two-dimensional Fourier transform on *H*

(3) $$H_{v}=FFT_{2D}\left[H\right].$$

Since the mm Wave channel is sparse in the angular domain, the (2) can be expressed as

(4) $$H=B_{r}H_{v}B_{t}^{H},$$

where $B_{r}\in C^{M\times M}$ and $B_{t}\in C^{U\times U}$ are the discrete Fourier transform (DFT) matrices. *U* users send orthogonal pilot $X\in C^{U\times s}$ to the BS where *s* represents the pilot length, and the received signal $R\in C^{M\times s}$ at the BS is given by

(5) $$R=\sqrt{P}HX+W,$$

where $W\in C^{M\times s}$ is a complex additive noise matrix subject to gaussian distribution, and *P* is the transmitted power of the pilot signal. Let $X=B_{t}F$, then the received signal at BS is

(6) $$\begin{array}{cc} R & =\sqrt{P}HX+W\\ \; & =\sqrt{P}B_{r}H_{v}B_{t}^{H}B_{t}F+W.\\ \; & =\sqrt{P}B_{r}H_{v}F+W \end{array}$$

Vectorization of the received signal *R*

(7) $$\begin{array}{cc} vec\left(R\right) & =vec\left(\sqrt{P}B_{r}H_{v}F+W\right)\\ \; & =\left(F^{T}⊗\sqrt{P}B_{r}\right)vec\left(H_{v}\right)+vec\left(W\right),\\ \; & =ah+\bar{w} \end{array}$$

where Equation (7) satisfies $vec\left(ABC\right)=\left(C^{T}⊗A\right)vec\left(B\right)$, $a=F^{T}⊗\sqrt{P}B_{r}$, $h=vec\left(H_{v}\right)$, $\overset{-}{w}=vec\left(W\right)$. ⊗ is the Kronecker product and the received signal is quantized by 1-bit ADC at BS

(8) $$\begin{array}{cc} \tilde{Y} & =\Gamma \left(vec\left(R\right)\right)\\ \; & =\Gamma \left(\left(F^{T}⊗\sqrt{P}B_{r}\right)vec\left(H_{v}\right)+vec\left(W\right)\right),\\ \; & =\Gamma \left(ah+\bar{w}\right) \end{array}$$

where $\Gamma \left(\cdot \right)$ is a function that quantizes the real and imaginary parts of the received signal, and the element in $\tilde{Y}$ comes from the set $Q\in \left\{1+j,1-j,-1+j,-1-j\right\}$

(9) Y∼=sgnRevecR+j·sgnImvecR=sgnReah+w_+j·sgnImah+w_,

where $\mathrm{sgn}\left(\cdot \right)$ is the signum function for one-bit quantization defined as

(10) $$\mathrm{sgn}\left(x\right)=\left\{\begin{array}{c} \begin{array}{ccc} 1 & \; & x\geq 0,\\ -1 & \; & x<0. \end{array} \end{array}\right.$$

Now write the complex signal in the form of a real signal

(11) Y∼=ResgnvecRImsgnvecR,h=RevecHvImvecHv.

(12) w_=RevecWImvecW,a=ReFT⊗PBr−ImFT⊗PBrImFT⊗PBrReFT⊗PBr.

## 3. Channel Estimation Based on Residual Learning and Multi-Path Feature Fusion

### 3.1. DL-Based Image Super-Resolution and Channel Estimation

The conventional compressed sensing-based channel estimation algorithm faces high-dimensional matrix inversion operations with the increase in antennas at the BS, which results in unsatisfactory performance at low SNR regions and requires huge pilot overhead. In this paper, we directly use the quantized received measurements $\tilde{Y}$ and the known pilot *X* to recover the mm Wave channel by a deep learning method. It is assumed that the BS is equipped with M=32 antennas to serve U=16 single-antenna users. At a certain moment, each user transmits a pilot sequence of length 8 to BS. $\tilde{Y}\in C^{32\times 8\times 2}$ and $X\in C^{16\times 8\times 2}$ can be regarded as low-resolution images with two channels, while $H\in C^{32\times 16\times 2}$ is a high-resolution image. To make full use of the correlation in the spatial arrangement of quantized measurements and target channel, we consider the real part and imaginary part as two dimensions of the same image. In the field of computer vision, recovering a high-resolution image from a low-resolution image is an important research problem that can be described as

(13) $$I_{HR}=\varsigma \left(I_{LR};\theta \right),$$

where $I_{LR}$ is the low-resolution image, $I_{HR}$ is the recovered high-resolution image, and ς is the deep learning model defined by parameter θ. With the help of the deep learning-based super-resolution, the estimated mm Wave channel can be expressed as

(14) $$\overset{\wedge }{H}=\Psi_{RL-MFF-N\mathrm{et}}\left(\tilde{Y},X;\Theta \right),$$

where $\overset{\wedge }{H}$ is the prediction and Θ is the parameter of the estimator $\Psi_{RL-MFF-Net}$. Our ultimate purpose is to obtain an estimator $\Psi_{RL-MFF-Net}$ that reconstructs the corresponding high-resolution counterpart $\overset{\wedge }{H}$ for given low-resolution $\tilde{Y}$ and *X* as input.

### 3.2. The Proposed RL-MFF-Net

**Figure 2:**
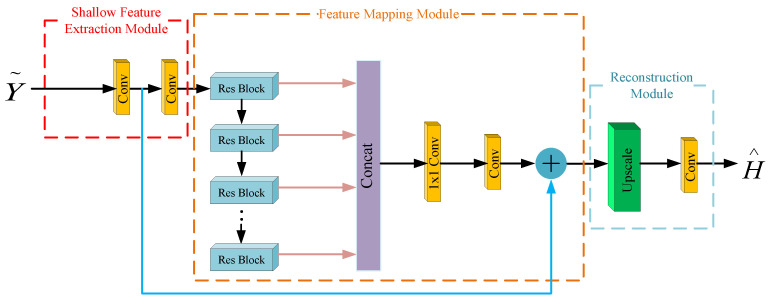
Architecture of the proposed RL-MFF-Net-based channel estimation.

The proposed RL-MFF-Net framework is depicted in Figure 2, which is composed of a shallow feature extraction module, feature mapping module and reconstruction module.

Since the pilot shows no change throughout our simulation, the quantized received measurements $\tilde{Y}$ are the input for the RL-MFF-Net. The features extracted from the convolutional layer of the shallow feature extraction module are

(15) $$G_{-2}=f_{2}\left(f_{1}\left(\tilde{Y}\right)\right)=f_{2}\left(G_{-1}\right),$$

where $f_{i}\left(\cdot \right)$ stands for the function of the convolutional layer, then the extracted mm Wave channel shallow feature $G_{-2}$ is sent to the feature mapping module for deep feature learning. The output of the *k*-th residual block is obtained by

(16) $$G_{k}=\Gamma_{k}\left(G_{k-1}\right)=\Gamma_{k}\left(\Gamma_{k-1}\left(\ldots \left(\Gamma_{1}\left(G_{-2}\right)\right)\ldots \right)\right),$$

where $\Gamma_{k}$ denotes the *k*-th residual block of the feature mapping module, $G_{k-1}$ is the input of the *k*-th block and $G_{k}$ is the corresponding output by fully utilizing the convolutional layers in the residual block. The mm Wave channel’s local features extracted from *K* residual blocks are preserved via multi-path global feature fusion

(17) $$\overset{-}{G}=f_{GF}\left(\left[G_{1},G_{2},\ldots ,G_{K}\right]\right),$$

where $\left[G_{1},G_{2},\cdots ,G_{K}\right]$ denotes the concatenation of the local feature maps, $\overset{-}{G}$ denotes the mm Wave channel global feature and $f_{GF}$ is a composite function of 1×1 and 3×3 convolution. To alleviate the vanishing-gradient problem of the RL-MFF-Net-based estimator, the long skip connection is further implemented in the estimator to prevent the model’s degradation

(18) $$G_{D}=G_{-1}+\overset{-}{G},$$

where $G_{-1}$, which is used for further shallow feature extraction and global residual learning, is the shallow feature extracted from the first convolution layer. After extracting the local and global features in the low-resolution space, the whole mm Wave channel is finally estimated through a reconstructed module

(19) $$\overset{\wedge }{H}=\Phi \left(G_{D}\right),$$

where Φ is the composite function consisting of an upscale layer and convolution layer.

The proposed RL-MFF-Net in this paper consists of eight residual blocks. In addition to the 1×1 convolution kernel that is applied for local feature fusion and global feature fusion, the 3×3 convolution kernel is utilized for feature extraction in all remaining convolutional layers. Zero-padding is used to guarantee that the size of the feature remains constant after convolution. Comparing with the traditional residual block in [29], dense connection and multi-path feature fusion are implemented to enable the current residual block can read the state of the previous block and preserve the hierarchical mm Wave channel features extracted from each convolution in the residual block. More details about the proposed residual block will be shown in Section 3.3.

### 3.3. Multi-Path Feature Fusion and Dense Connection

**Figure 3:**
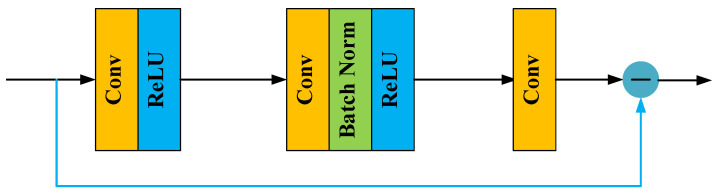
The network structure of the DnCNN proposed in [16].

Motivated by advantages of residual learning, the authors of [16] proposed a denoising convolutional neural network (DnCNN) for channel amplitude estimation, and Figure 3 illustrates the network architecture of the DnCNN.

**Figure 4:**
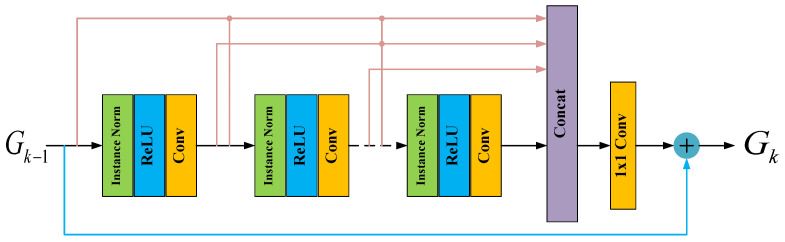
Residual block based on multi-path feature fusion and dense connection.

Although DnCNN outperforms other DL-based estimation methods, it neglects to fully use features extracted from each convolutional layer. Inspired by the densely connected network [30] and feature pyramid network (FPN) [31], we propose a residual block based on multi-path feature fusion and dense connection as the basic block for the feature mapping module, which is shown in Figure 4.

Each residual block is comprised of an instance normalization layer [32], ReLU layer and convolutional layer. The instance normalization is able to accelerate the convergence of the estimator. In order to make full use of the underlying features from the quantized measurements for accurate reconstruction of the mm Wave channel, we conduct a dense connection allowing the output of the k−1-th residual block to directly access each layer of the *k*-th block. Meanwhile, the features extracted from each convolutional layer in the current block access all the subsequent layers and we pass on mm Wave channel features that need to be preserved. After concatenating the states of all the layers within the current block, we further conduct multi-path local feature fusion to adaptively preserve the underlying mm Wave channel features for local residual learning.

Denoting $G_{k-1}$ and $G_{k}$ to be the input and output of the *k*-th residual block, we have

(20) $$G_{k}=G_{k-1}+G_{k,LF},$$

where $G_{k}$ and $G_{k-1}$ denote feature maps extracted from the current and preceding residual block, respectively, and $G_{k,LF}$ refers to the underlying feature preserved by using multi-path local feature fusion

(21) $$G_{k,LF=}f_{LF}^{k}\left(\left[G_{k-1},G_{k,1},G_{k,2},\ldots ,G_{k,M}\right]\right),$$

where $\left[G_{k-1},G_{k,1},G_{k,2},\cdots ,G_{k,M}\right]$ denotes the concatenation of the mm Wave channel future extracted from the previous residual block and the whole convolution layer in current residual block. $f_{LF}^{k}$ is defined as the 1×1 convolution operation in the *k*-th residual block for adaptive control feature fusion. $G_{k,m}$ is the output of the *m*-th convolution layer in the current block, which can be written as

(22) $$G_{k,m}=\zeta \left(W_{k,m}\left[G_{k-1},G_{k,1},G_{k,2},\ldots ,G_{k,m-1}\right]\right),$$

ζ is the ReLU activation function, and $W_{s,m}$ is the weight of the *m*-th layer.

## 4. Learning Strategy for Channel Estimation and Dataset Generation

### 4.1. Learning Strategy for Channel Estimation

In this paper, the RL-MFF-Net-based channel estimator mainly works in an offline training phase and online deployment phase. In the offline training phase, a training set is given as

(23) $$\left({\tilde{Y}}_{train},H_{train}\right)=\left\{\left({\tilde{Y}}_{1},H_{1}\right),\ldots ,\left({\tilde{Y}}_{N},H_{N}\right)\right\},$$

where $\left({\tilde{Y}}_{n},H_{n}\right),n\in \left\{1,2,\ldots ,N\right\}$ denotes the *n*-th training example of the set. ${\tilde{Y}}_{n}\in C^{M\times s\times 2}$ is the input and $H_{n}\in C^{M\times U\times 2}$ is the label. Our goal is to optimize overall trainable variables by minimizing the mean of squared errors (MSE) as follows

(24) $$L\left(\theta \right)=\frac{1}{N}\sum_{n=1}^{N}{\left(\Psi_{RL-MFF-Net}\left({\tilde{Y}}_{n};\Theta \right)-H_{n}\right)}^{2},$$

where $\Psi_{RL-MFF-Net}\left(\cdot \right)$ denotes the estimator parameterized by Θ, ${\tilde{Y}}_{n}$ is the input of the estimator and $H_{n}$ represents the ground truth. The other hyper-parameters are summarized in Table 1.

The $L\left(\Theta \right)$ can be regarded as a function of the estimator parameters Θ, we adopt the adaptive moment estimation (ADAM) algorithm [33] to optimize the loss function based on the proposed RL-MFF-Net framework. The ADAM iteration can be written as

(25) $$\Theta^{j+1}=\Theta^{j}-\alpha \frac{{\overset{\wedge }{M}}_{j}}{\sqrt{{\overset{\wedge }{V}}_{j}}+\varepsilon },$$

where *j* is the timestep and $\Theta^{0}$ is represented as the initial parameter. α is the step size and ε is used to ensure that the denominator is greater than zero. ${\overset{\wedge }{M}}_{j}$ and ${\overset{\wedge }{V}}_{j}$ are the corrections to the first-order moment estimate $M_{j}$ and second-order moment estimate $V_{j}$, respectively.

(26) $${\overset{\wedge }{M}}_{j}=\frac{M_{j}}{1-\beta_{1}^{j}},$$

(27) $${\overset{\wedge }{V}}_{j}=\frac{V_{j}}{1-\beta_{2}^{j}},$$

where $\beta_{1}$ and $\beta_{2}$ are the decay rates for the first-order and second-order moment estimate, respectively. Specifically, $\beta_{1},\beta_{2}\in \left[0,1\right)$. The update equations for $M_{j}$ and $V_{j}$ are as follows

(28) $$M_{j}=\beta_{1}\cdot M_{j-1}+\left(1-\beta_{1}\right)\cdot g_{j},$$

(29) $$V_{j}=\beta_{2}\cdot V_{j-1}+\left(1-\beta_{2}\right)\cdot g_{j}^{2},$$

where $g_{j}$ refers the gradient of the loss function. In the online deployment phase, by putting the test data ${\tilde{Y}}_{test}$, the trained RL-MFF-Net-based estimator can directly reconstruct the mm Wave channel $\overset{\wedge }{H}$. The proposed RL-MFF-Net-based algorithm is summarized in Algorithm 1, where *G* is the maximum iteration number.
**Algorithm 1 ** RL-MFF-Net-based channel estimation algorithm**offline training phase:** 1:Initialize j=0, estimator parameters Θ, stepsize α, learning rate and decay rate. 2:**Input: ***Training set $\left({\tilde{Y}}_{train},H_{train}\right)$* 3:**while**j≤G**do** weights update 4:$g_{j}\leftarrow \nabla_{\Theta }L_{j}\left(\theta_{j-1}\right)$ 5:Compute $M_{j}$ and $V_{j}$ by Equation (27) and (28) 6:Computer ${\overset{\wedge }{M}}_{j}$ and ${\overset{\wedge }{V}}_{j}$ by Equation (25) and (26) 7:Update estimator parameters $\Theta^{j+1}\leftarrow \Theta^{j}-\alpha \frac{{\overset{\wedge }{M}}_{j}}{\sqrt{{\overset{\wedge }{V}}_{j}}+\varepsilon }$ 8:j=j+1 9:**end while** 10:**Output: ***Well-trained estimator $\Psi_{RL-MFF-Net}\left(\cdot \right)$***online deployment phase:** 11:**Input: ***Test set ${\tilde{Y}}_{test}$* 12:**do** *Channel Estimation with $\Psi_{RL-MFF-Net}\left(\cdot \right)$* 13:**Output: ***the reconstructed mm Wave channel $\overset{\wedge }{H}=\Psi_{RL-MFF-Net}\left({\tilde{Y}}_{test}\right)$*

### 4.2. Dataset Generation

In order to train the RL-MFF-Net-based estimator, it is necessary to obtain channel datasets and quantized received measurement datasets. The channels between the BS and users are generated by using the publicly-available generic DeepMIMO dataset [34]. The DeepMIMO is defined by the parameters set and ray-tracing scenario. Based on the setup of the channel parameters as in Table 2, we can construct the channel samples between the BS and the users according to (1) and (2) and quantized received measurement samples according to (5)–(11). Specifically, we generate four different channel matrix $H_{k}$ with the size of 16×8, 32×16, 64×32, and 128×64, respectively. We use 60% of the datasets for training, 30% for testing and 10% for validation.

## 5. Simulation Results

In this section, the RL-MFF-Net-based channel estimation algorithm is compared with other DL-based methods and the traditional algorithm GAMP. We investigate the performance of the estimator with the metric of MSE as

(30) $$MSE=\frac{1}{M}\sum_{k=1}^{M}{∥{\overset{\wedge }{H}}_{k}-H_{k}∥}^{2},$$

where *M* is the number of test samples, and $H_{k}$ and ${\overset{\wedge }{H}}_{k}$ are the target channel and the predicted value of the proposed estimator, respectively.

**Figure 5:**
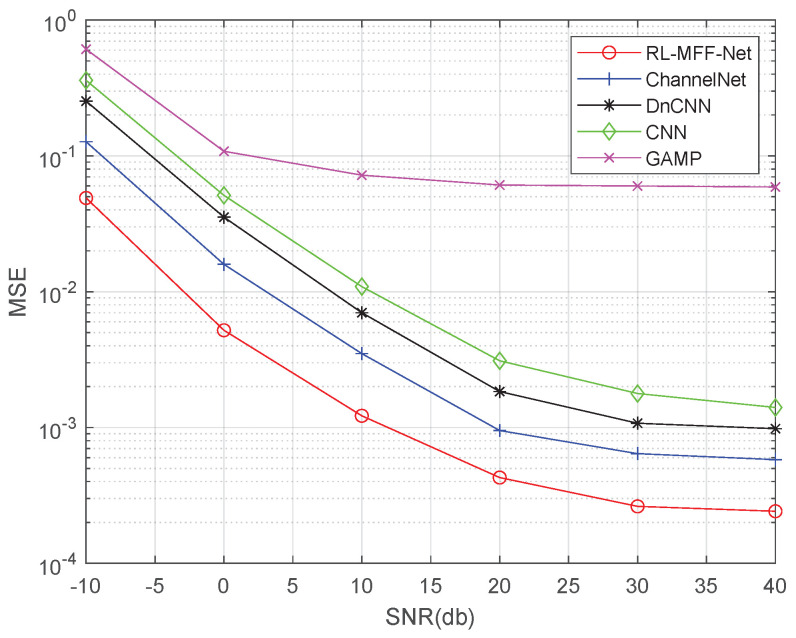
The comparison of channel estimation performance for different methods.

Figure 5 shows the MSE performance comparison of the ChannelNet, DnCNN, CNN, GAMP and the proposed RL-MFF-Net. In our simulations, we consider that the BS equips M=32 antennas to serve U=16 single-antenna users. The number of pilots is set to s=8. As shown in Figure 5, the system in four DL-based algorithms achieves better MSE performance than that of the conventional GAMP algorithm. In particular, the proposed RL-MFF-Net outperforms the other mentioned DL-based methods in all considered SNR regions. Moreover, RL-MFF-Net persists to achieve 4 dB gains over the ChannelNet especially in the low SNR region due to the joint use of residual learning, multi-path feature fusion and dense connection.

**Figure 6:**
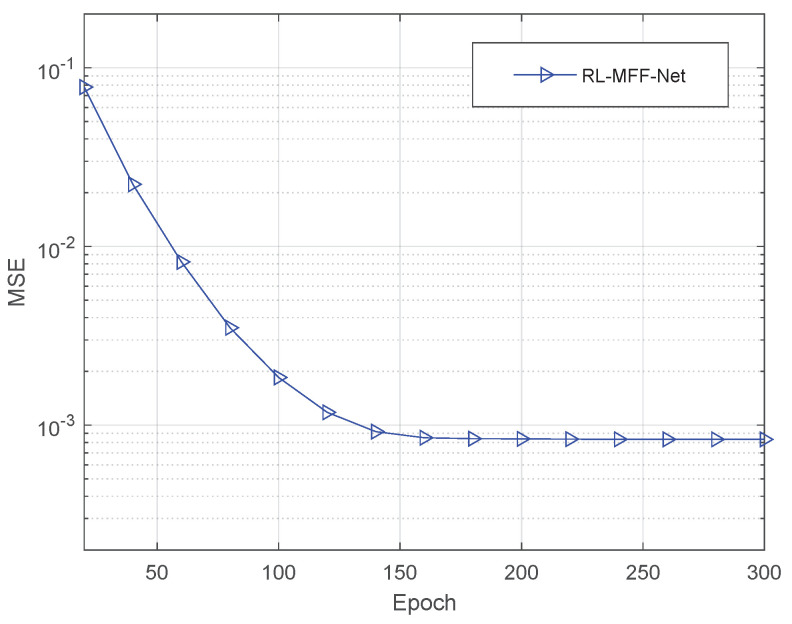
Convergence performance of the proposed RL-MFF-Net-based channel estimation algorithm.

Figure 6 shows the convergence performance versus the number of training epochs with the proposed RL-MFF-Net-based algorithm in which the SNR is 10 dB. We can observe that the convergence of the scheme improves as training epochs increase. During RL-MFF-Net training, the MSE curve becomes stable after around 150 of training epochs.

### 5.1. Impact of System Parameters

In this subsection, we show how the MSE performance of the proposed RL-MFF-Net-based method changes for the variation of the system parameters.

**Figure 7:**
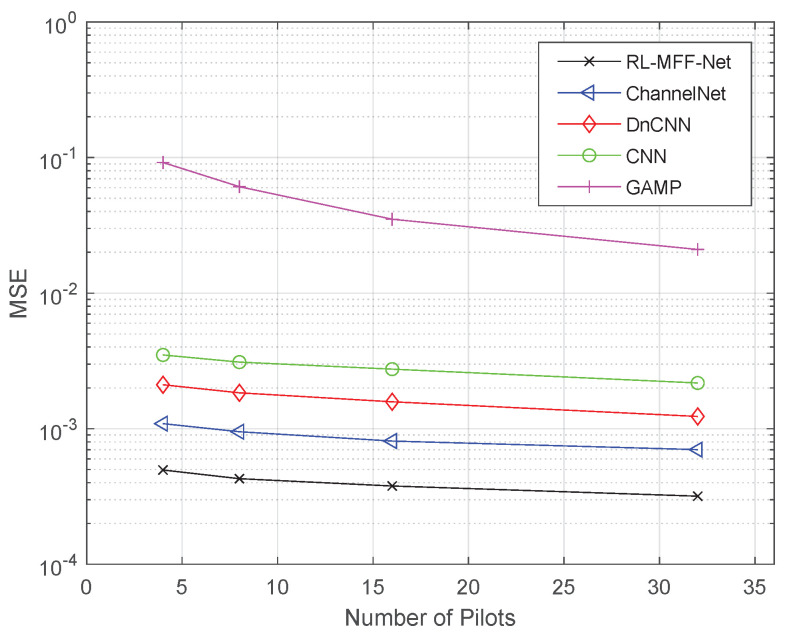
The MSE performance comparison of different methods versus the number of pilots.

Figure 7 shows the MSE performance comparison for the five channel estimation methods with respect to a different number of pilots. It is noticed that the RL-MFF-Net-based estimator achieves much higher estimation accuracy than other schemes with only a small number of pilots, which greatly reduces pilot overhead for the mm Wave massive MIMO system. For the number of pilots s=32, the proposed method achieves 3 dB gains compared with the ChannelNet.

**Figure 8:**
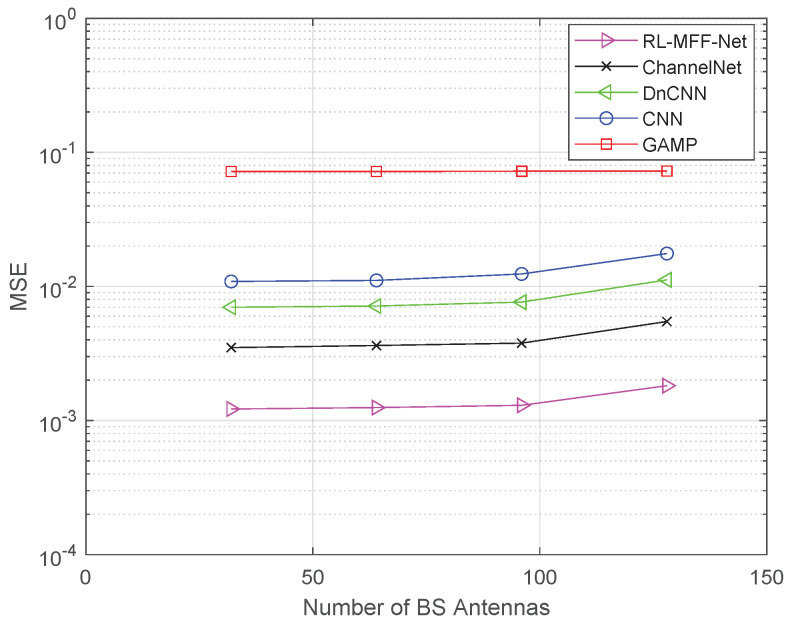
The MSE performance comparison of different methods versus the number of BS antennas.

Figure 8 shows the MSE performance of five different methods versus the number of BS antennas. It can be seen that the four DL-based schemes achieve better performance than the traditional GAMP algorithm. In particular, the proposed RL-MFF-Net-based estimator enjoys lower estimation error as the number of BS antennas increase. Moreover, the proposed method still outperforms ChannelNet 4 dB gains even at M=128 antennas.

**Figure 9:**
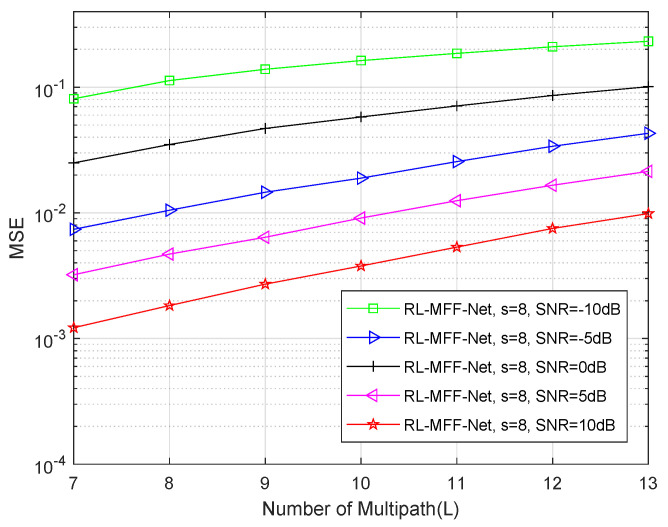
The MSE performance for the proposed RL-MFF-Net-based scheme with different multi-paths.

We further investigate the robustness of the proposed RL-MFF-Net-based channel estimation method as a function of the number of multi-path L in Figure 9. Noting that the proposed RL-MFF-Net-based estimator mainly works two different phases, the multi-path components is set to L = 10 during the offline training phase. However, it can be concluded that the proposed method robustly estimates the channels path with L≠10 at the online deployment phase.

### 5.2. Impact of Hyper Parameters

To determine the best estimator structure for mm Wave massive MIMO system channel estimation, we investigate the impact of hyper parameters on estimator performance. Here, the BS is equipped with 32 antennas to serve 16 single-antenna users and the number of pilot is set as 8.

**Figure 10:**
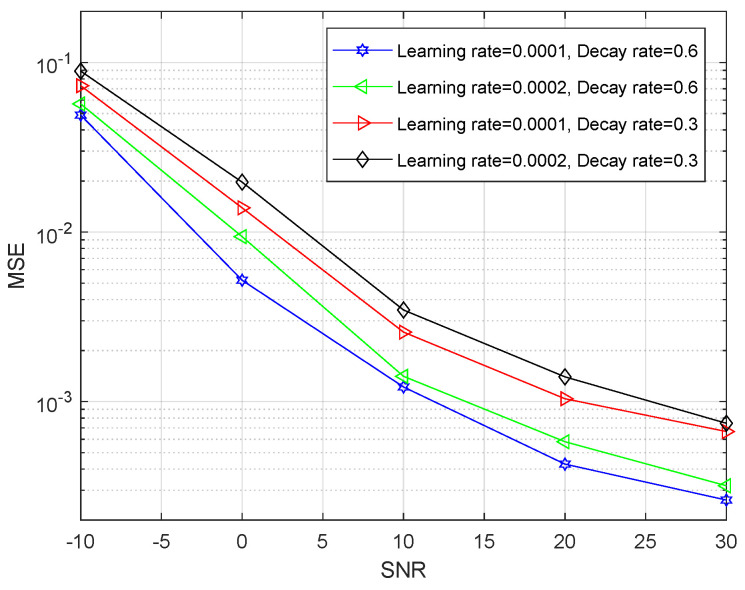
The MSE performance of the proposed RL-MFF-Net-based scheme when the learning rate and the decay rate are different.

Figure 10 shows that the MSE value versus the SNR with the RL-MFF-Net-based estimation algorithm in which the learning rate and decay rate are different. It can be clearly seen that the MSE performance in the case of “Learning rate = 0.0001, Decay rate = 0.6” outperforms that of other cases in terms of SNR, which implies that introducing a smaller learning rate and larger decay rate can boost the performance of the channel estimation based on the proposed method. However, too small a learning rate will lead to converge slowly while too large a decay rate will make the loss function pass by a global minimum point, which means that selecting an appropriate learning rate and decay rate is a significant issue for improving the MSE performance of the RL-MFF-Net-based channel estimation.

**Figure 11:**
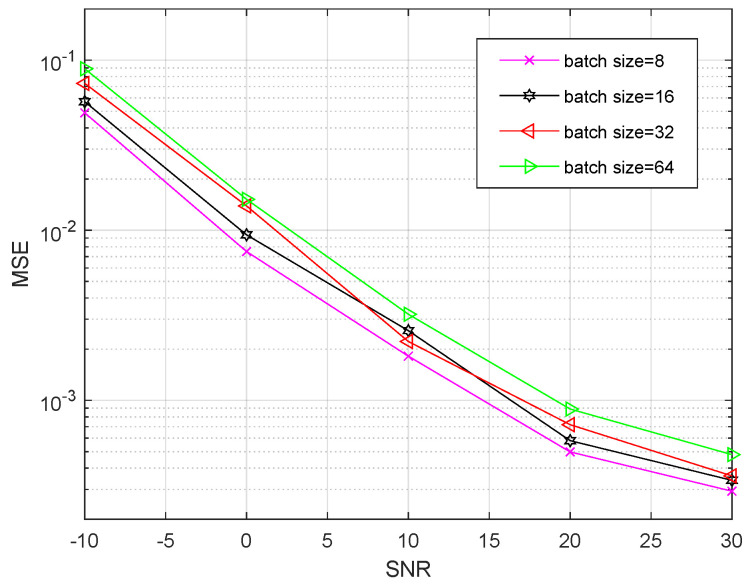
The MSE performance of the proposed RL-MFF-Net-based scheme with different batch size.

Figure 11 shows that the MSE performance of our proposed RL-MFF-Net-based channel estimation scheme for different batch size is a function of SNR. It is shown that the MSE of the channel estimation is reducing with the increasing SNR. Meanwhile, The simulation results show that the MSE performance in the case of “batch size = 8” achieves a more satisfactory performance than other cases, which implies that it is better to choose an appropriate batch size during the offline training phase.

**Figure 12:**
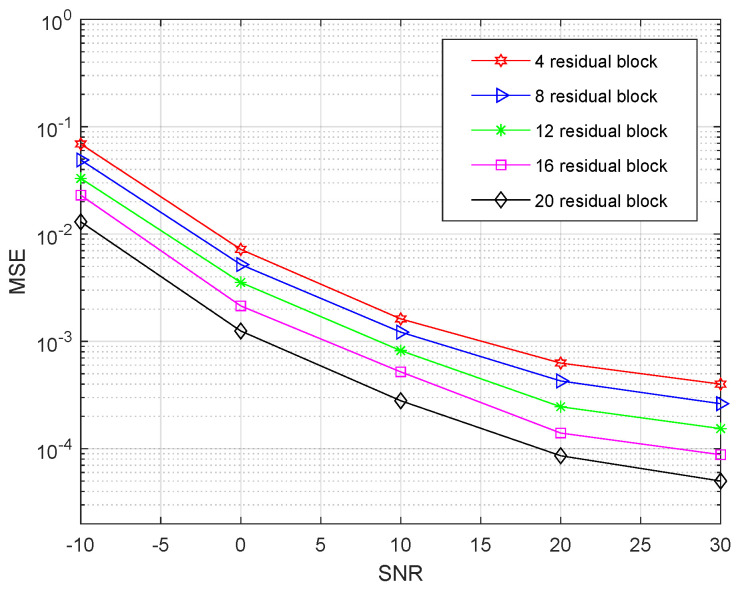
The MSE performance of the proposed RL-MFF-Net-based scheme with a different number of residual blocks.

Figure 12 investigates the MSE performance of the proposed RL-MFF-Net-based channel estimation method with different residual blocks. The results show that the estimator’s MSE improves with the increasing number of residual blocks. However, simply stacking residual blocks to construct deeper networks for channel estimation is more difficult to train. The reason for better performance is that it exploits dense connection to ensure maximum information flow between the layers of the estimator. Meanwhile, multi-path feature fusion further allows the estimator to make full use of the hierarchical features from the quantized received measurements. At the SNR value of 20 dB, the RL-MFF-Net with 20 residual blocks provides around 6 dB gains over the four residual blocks.

### 5.3. Comparison of the Traditional Residual Block and Residual Block Based on Multi-Path Feature Fusion with Dense Connection

In order to verify the effectiveness of the proposed multi-path feature fusion and dense connection-based residual block, we further compare the MSE performance of the estimator with a traditional residual block in [29] and the proposed residual block, as shown in Figure 4.

**Figure 13:**
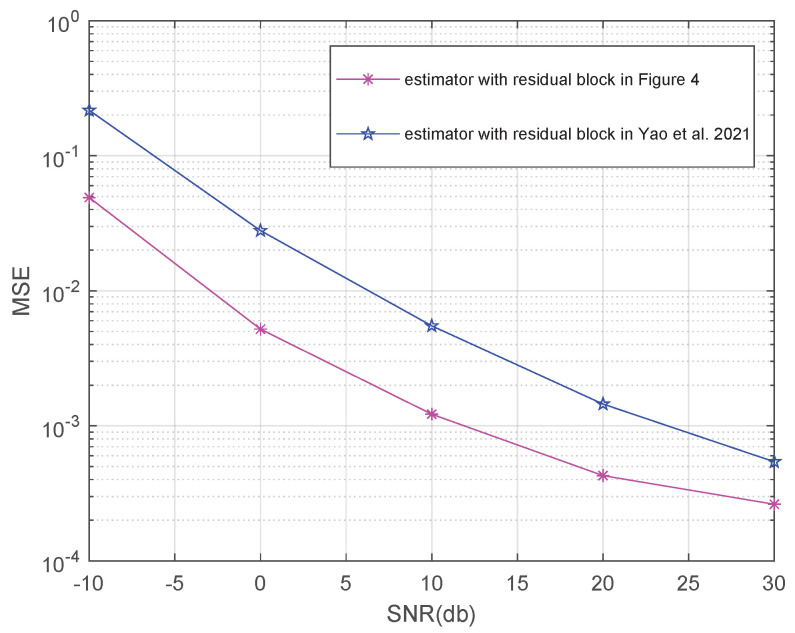
The MSE performance of the RL-MFF-Net with traditional residual blocks and the proposed residual blocks. Estimator with residual block in Yao et al. 2021 [20].

It is obvious from Figure 13 that the proposed residual block based on multi-path feature fusion and dense connection is able to greatly improve the MSE performance of the estimator compared with the traditional residual block, especially in the low SNR region. It is due to multi-path feature fusion, and the dense connection can make full use of the underlying features from quantized received measurements for accurate reconfiguration of the mm Wave channel.

### 5.4. Impact of Residual Learning, Multi-Path Fusion and Dense Connection

Table 3 shows the ablation investigation on the effects of residual learning (RL), multi-path feature fusion (MFF) and dense connection (DC) on the channel estimator.

We find that the baseline is obtained without RL, MFF, or DC and performs poorly (MSE = $0.97\times 10^{-2}$), which is mainly caused by the difficulty of training and fails to make full use of the hierarchical features from the quantized received measurements. When one of RL, MFF, or DC is added to the baseline, each component can improve the MSE performance of the baseline. Furthermore, the baseline with two components performs better than with only one component. It is obvious that we obtain the optimal channel estimation performance while using three components simultaneously.

## 6. Conclusions

In this paper, we propose a novel RL-MFF-Net-based channel estimation method for a mm Wave massive MIMO system. Specifically, we regard the quantized received measurements at the BS as a low-resolution image and use a DL-based image super-resolution technique to reconstruct the mm Wave channel accurately. Initially, we introduce residual learning to train only the high frequency residual part between the quantized received measurements and target the mm Wave channel for reducing the training difficulty of the channel estimator. Moreover, we conduct dense connection to address the gradient dispersion problem of the estimator due to stacking residual blocks. Finally, we employ multi-path feature fusion to make full use of the underlying features extracted from the quantized received measurements. For future work, we will apply the proposed RL-MFF-Net-based estimator to address the channel estimation problem in a terahertz (THz) communication system.

## Figures and Tables

**Table 1:** Simulation parameters.

Parameter	Value
Learning rate	0.0001
Decay rate	0.6
Optimizer	ADAM
Batch size	8
Training epoch	300
Loss function	$L_{2}$
Number of residual blocks	4∼20

**Table 2:** The deep MIMO dataset parameters.

Parameter	Value
Carrier frequency	28 GHz
System bandwidth	100 MHz
Active BSs	32
Active users	From row R1 to R502
Number of BS antennas	$\left(M_{x},M_{y},M_{z}\right)=\left(1,16,1\right);\left(1,32,1\right);\left(1,64,1\right);\left(1,128,1\right)$
Number of user antennas	$\left(M_{x},M_{y},M_{z}\right)=\left(1,1,1\right)$
Antenna space	0.5
The length of pilot	4∼32
Number of paths	10

**Table 3:** Different combinations of RL, MFF, and DC.

Experiments	RL	MFF	DC	MSE
1	*√*	*√*	*√*	$0.12\times 10^{-2}$
2	*√*	*√*	×	$0.42\times 10^{-2}$
3	*√*	×	*√*	$0.49\times 10^{-2}$
4	×	*√*	*√*	$0.44\times 10^{-2}$
5	*√*	×	×	$0.71\times 10^{-2}$
6	×	*√*	×	$0.69\times 10^{-2}$
7	×	×	*√*	$0.73\times 10^{-2}$
8	×	×	×	$0.97\times 10^{-2}$

## Data Availability

Not applicable.
